# Radiologic Mimics of Osteomyelitis and Septic Arthritis: A Pictorial Essay

**DOI:** 10.1155/2021/9912257

**Published:** 2021-05-24

**Authors:** Wanyin Lim, Christen D. Barras, Steven Zadow

**Affiliations:** ^1^Dr Jones and Partners Medical Imaging, Adelaide, South Australia, Australia; ^2^Department of Radiology, Royal Adelaide Hospital, Adelaide, South Australia, Australia; ^3^South Australian Health and Medical Research Institute and the University of Adelaide, Adelaide, South Australia, Australia; ^4^Flinders Medical Centre, Bedford Park, South Australia, Australia

## Abstract

Various imaging techniques may be employed in the investigation of suspected bone and joint infections. These include ultrasound, radiography, functional imaging such as positron emission tomography (PET) and nuclear scintigraphy, and cross-sectional imaging, including computed tomography (CT) and magnetic resonance imaging (MRI). The cross-sectional modalities represent the imaging workhorse in routine practice. The role of imaging also extends to include assessment of the anatomical extent of infection, potentially associated complications, and treatment response. The imaging appearances of bone and joint infections are heterogeneous and depend on the duration of infection, an individual patient's immune status, and virulence of culprit organisms. To add to the complexity of radiodiagnosis, one of the pitfalls of imaging musculoskeletal infection is the presence of other conditions that can share overlapping imaging features. This includes osteoarthritis, vasculopathy, inflammatory, and even neoplastic processes. Different pathologies may also coexist, for example, diabetic neuropathy and osteomyelitis. This pictorial review aims to highlight potential mimics of osteomyelitis and septic arthritis that are regularly encountered, with emphasis on specific imaging features that may aid the radiologist and clinician in distinguishing an infective from a noninfective aetiology.

## 1. Introduction

Osteomyelitis is defined as bone inflammation due to infection whilst septic arthritis is defined by infection within a joint [1]. Bone and joint infections have a variable clinical presentation ranging from acute sepsis to insidious onset of pain, with or without fever. Laboratory results such as white cell count, C-reactive protein, and erythrocyte sedimentation rate are often, but not always, abnormal. In some cases, blood cultures are positive. Ultimately, bone biopsy may ultimately be required to identify the offending organism [2].

Imaging plays a crucial role in the diagnosis of infection, assessing disease extent, associated complications, planning biopsy sites, and monitoring treatment response. The various imaging tools employed include radiography, ultrasound, computed tomography (CT), and functional studies including nuclear scintigraphy, white cell scan, and positron emission tomography (PET). Magnetic resonance imaging (MRI), being moderately sensitive and specific, is the imaging modality of choice in the evaluation of suspected osteomyelitis [3, 4].

Understandably, given the heterogeneity of clinical presentations, imaging appearances of infection also vary considerably, being affected by factors such as the virulence of the implicated organism, the site of involvement, the individual's immune status, and the duration of symptoms [1]. To add to the complexity, imaging features for infection are not necessarily specific for osteomyelitis and can also be shared with multiple other conditions [1]. These include degenerative processes, trauma or stress responses, neoplastic processes, vasculopathy, inflammation, neuropathic arthropathy, iatrogenic processes, and depositional diseases. At times, clinical details may be insufficient to aid image interpretation.

This pictorial review aims to highlight a few of the potential mimics of osteomyelitis and septic arthritis that are commonly encountered, with emphasis on specific MRI features that may aid the radiologist and clinician in distinguishing infection from a noninfective aetiology. Variants of infection/osteomyelitis are also briefly covered.

### 1.1. Typical Imaging Appearances of Osteomyelitis and Septic Arthritis

**Figure 1:**
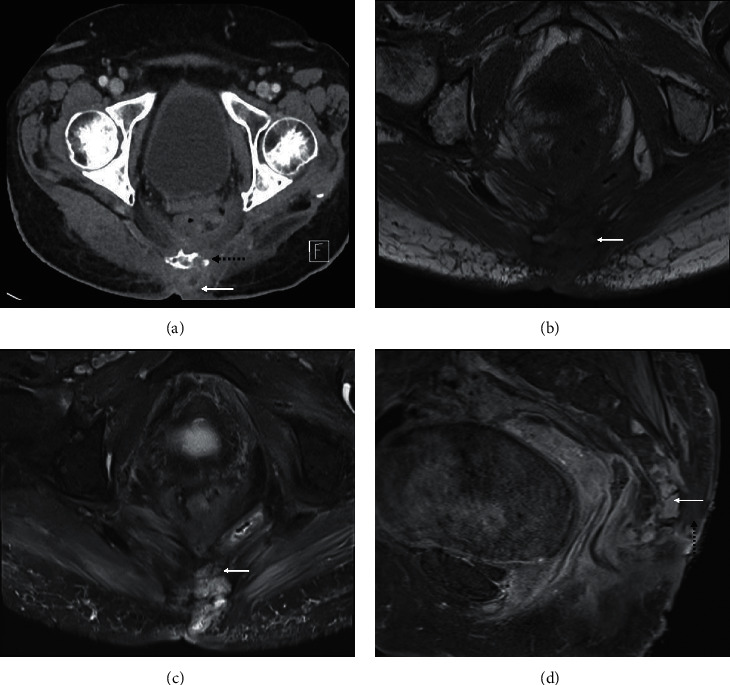
Osteomyelitis with an ulcer tract arising from a necrotic, infected pressure sore. Axial CT pelvis with intravenous contrast (a) of a 78-year-old bedbound female worked up for sepsis secondary to an infected pressure ulcer showed the ulcer extending to the coccyx in the left parasagittal region (arrow), with bone erosion (dotted line). The affected bone is hypointense on the axial T1 (b) and hyperintense on fluid-sensitive, PDFS (proton density with fat saturation, (c)) sequences. Sagittal T1FS postcontrast image (d) shows enhancement of the coccyx (arrow) and sacrococcygeal joint confirming the diagnosis. The adjacent deep subcutaneous region of nonenhancement is compatible with the area of necrosis (dotted arrow).

To understand the MR imaging appearances of infection mimics, it is first important to know the classic appearances of infection. Pyogenic osteomyelitis is typically T1 hypointense, due to the replacement of normal fatty marrow tissue. The confluent T1-hypointensity usually results in loss of distinction between the cortex and medulla, referred to as the “ghost sign” (Figure 1). On T2-weighted sequences, such as T2 with fat saturation (T2FS) and short tau inversion recovery (STIR), affected areas are fluid-hyperintense. Various terms are applied to this finding, including “marrow oedema,” “marrow oedema signal,” and “bone marrow lesion (BML)” [5]. Areas of established infection usually demonstrate enhancement following contrast administration.

**Figure 2:**
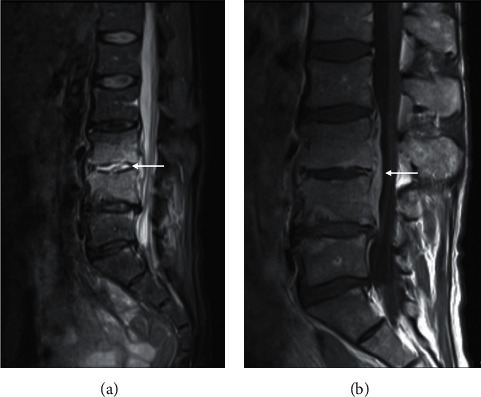
Discitis-osteomyelitis. (a) Sagittal STIR (short tau inversion recovery) with contiguous marrow oedema signal in the L3 and L4 vertebral bodies with involvement and effusion of the intervening L3/4 disc (arrow). Adjacent diffusely enhancing phlegmon (arrow) on a sagittal T1 postcontrast sequence (b) encroaching on the anterior epidural space, resulting in moderate spinal canal stenosis.

In peripheral body parts, ancillary features to actively search for include soft tissue inflammation, abscess, and ulceration or sinus tract formation, contiguous with the site of bone signal abnormality. Areas of geographical nonenhancement, which can indicate necrosis, are also important to note (Figure 1). In the spine, the involvement of two contiguous vertebral bodies and their intervening disc is usually indicative of discitis-osteomyelitis (Figure 2).

**Figure 3:**
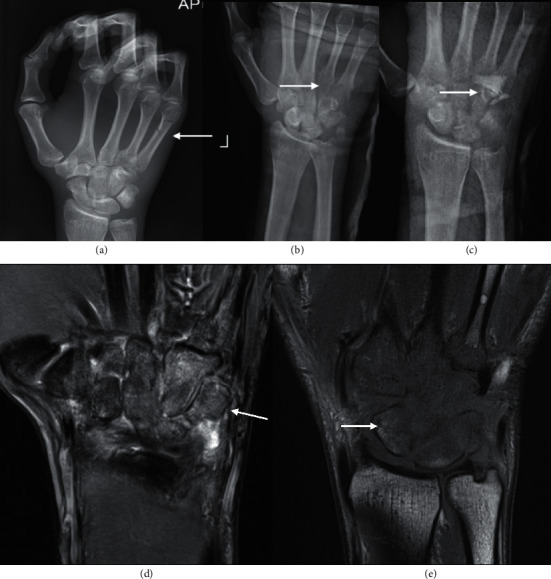
A 45-year-old man involved in a motor vehicle accident sustained an undisplaced 5^th^ metacarpal shaft fracture ((a), arrow). Repeating X-ray 16 days later (b), given the persistent pain, revealed rarefaction of 4^th^ and 5^th^ carpometacarpal (CMC) joints consistent with osteomyelitis and septic arthritis (arrow). X-ray-following washout and introduction of antibiotic-impregnated cement spacer (arrow). Coronal STIR (d) and T1 (e) sequences of the wrist show the extent of the infection involving all carpal bones and base of the 2^nd^ to 5^th^ metacarpals. (c)

The earliest findings of marrow oedema signal can be present within 2 days of symptom onset [1]. In the event of discordant clinical and imaging information, where there is a high clinical index of suspicion for infection despite negative initial imaging results, repeat imaging is important as imaging findings may lag behind clinical manifestations by up to 2 weeks [6] (Figure 3).

**Figure 4:**
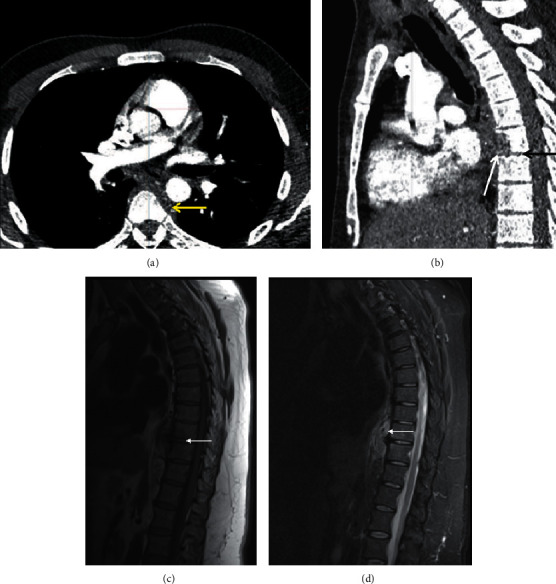
Tuberculous osteomyelitis. Postcontrast axial (a) and sagittal (b) chest CT of a 34-year-old patient with biopsy-proven TB discitis-osteomyelitis. There is lytic destruction of the T7 vertebral body anteriorly ((b) white arrow). There is subligamentous soft tissue density extending from T5-T8 (A yellow and B white arrows) with relative sparing of the adjacent T7 end plates (b black arrow). Sagittal T1 (c) and STIR (d) of a different patient with T9/10 osteomyelitis (c arrow) and pronounced subligamentous spread of disease from T8/9-T11 (d arrow). Note the relative sparing of the disc in early disease (c arrow).

With atypical organisms such as tuberculous osteomyelitis, systemic manifestations may not be as florid. Useful features for mycobacterial infection particularly in the spine are the relative sparing of intervertebral disc spaces early in the infective process, the subligamentous spread of infection, and involvement of multiple contiguous levels at once [1] (Figure 4).

**Figure 5:**
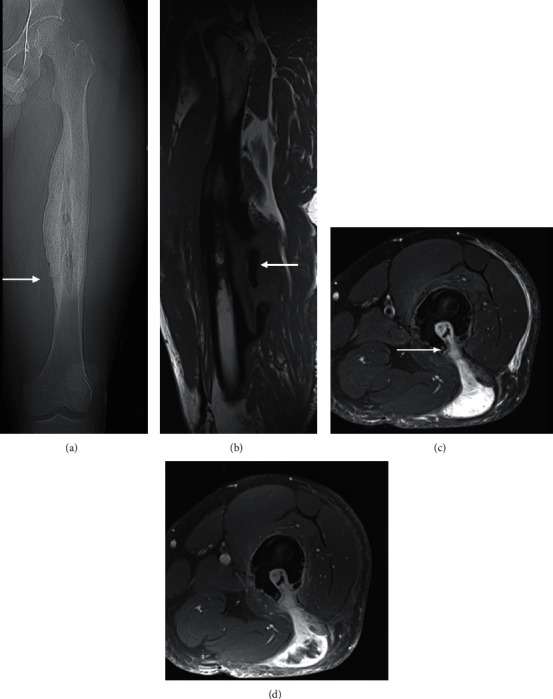
A 30-year-old systemically well man with chronic osteomyelitis who initially presented with an enlarging mass in the posterior thigh. The planning CT (a) showed femoral shaft sclerosis, periosteal thickening, and cortical expansion which is worse medially. A lucent tract at the inferior border of the abnormal bone extends from the medulla to the cortex, consistent with a sinus tract (arrow). Sagittal T1 (b) demonstrating the extent of the osteomyelitis with sequestrum posteroinferiorly (arrow). Axial PDFS (c) and T1FS postcontrast (d) MRI shows the new bone formation or involucrum attempting to envelop the infection. A cloaca or sinus tract involving the posterolateral thigh discharges into the subcutaneous plane, accounting for the presentation (C arrow).

Chronic osteomyelitis can occur in the context of incomplete treatment or atypical organisms. Disruption of vascular supply results in necrosis or sequestrum formation which then serves as a nidus for continued infection. To wall off the infection, granulation tissue and new bone, or involucrum, form around the sequestrum. Imaging is characterised by a mixed pattern of lysis and sclerosis [1]. Sinus tracts can form within the involucrum through which pus or sequestrum may be expelled (Figure 5).

**Figure 6:**
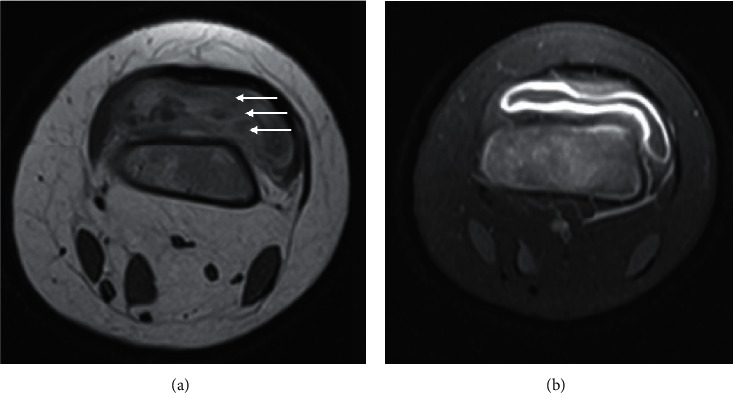
Axial T2 (a) and T1FS postcontrast (b) MRI of the knee in a 6-year-old boy. Lamellated synovial thickening (a) is specific for septic arthritis.

Features that are specific for joint infection include lamellated synovial thickening (Figure 6), rapid destruction, and erosion of the bare areas, where there is less coverage of the bone by hyaline cartilage [7]. Usually, there is a marrow oedema signal on both sides of the articular surface, often with an accompanying joint effusion and synovial enhancement, although these features are not as specific [7] (Figure 3).

### 1.2. Mimics of Osteomyelitis

#### 1.2.1. Degeneration/Mechanical Type

**Figure 7:**
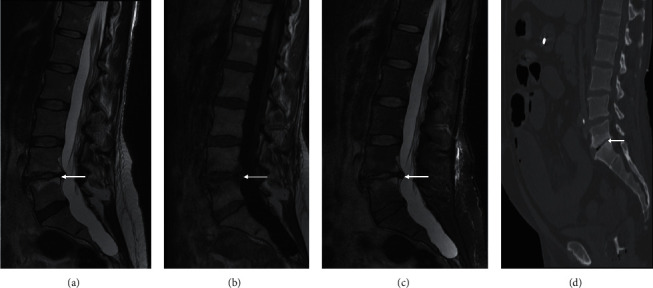
End plate degeneration. Sagittal T2 (a), T1 (b), and STIR (c) lumbar spine of a 40-year-old female presenting with right L5 radiculopathy in the background of low back pain. The fluid-hyperintensity (a, c) and T1 hypointense marrow signal (b) on either side of the L4/5 end plate (arrow), in the context of end plate irregularity and preservation of normal/hypointense disc signal, is consistent with inflammatory or Modic type I degeneration. The radiculopathy was secondary to impingement of the right L5 nerve root from the herniated disc at this level (not shown). Sagittal CT lumbar spine of a different patient (d) shows a vacuum cleft in the L5/S1 disc space with adjacent end plate sclerosis (Modic III degeneration). The vacuum cleft is a useful sign of degeneration.

Degenerative spondyloarthropathy, involving end plate degeneration in the spine, particularly of the inflammatory type (Modic type I), characterised by end plate T1-hypointensity and fluid-hyperintensity, can mimic infection. A useful distinguishing feature is end plate irregularity without erosion and presence of gas in the adjacent disc space, hypointense on all MRI sequences (Figure 7). The gas indicates the vacuum phenomenon in the context of degeneration [8, 9] (Figure 7(d)). The disc desiccation that accompanies degeneration is hypointense instead of hyperintense, as demonstrated in discitis [9].

**Figure 8:**
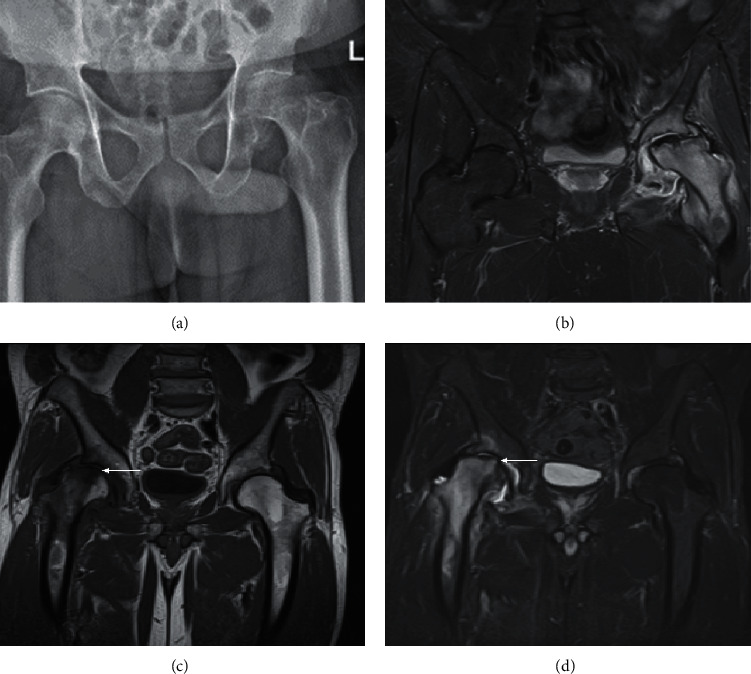
Two cases of rapidly progressive hip osteoarthritis. X-ray (a) and coronal STIR (b) of a 78-year-old man presenting with an increasingly painful and stiff left hip, demonstrating left hip joint effusion, joint marrow oedema signal, and flattening of the femoral head. Joint aspirate and subsequent washout were negative for infection. Coronal T1 (c) of a second patient with coronal STIR (d) performed 2 months apart shows the rapidly progressive nature of this condition, with hatchet-like deformity of the femoral head in the latter study (d arrow) due to subchondral insufficiency fracture.

Rapidly destructive osteoarthritis of the hip is a unique condition characterised by rapid cartilage loss [10]. The MR features include joint effusion, bone marrow oedema in the proximal femur and adjacent acetabulum, and hatchet-like deformity or flattening of the femoral head (Figure 8). These changes can mimic septic arthritis. Useful discriminatory features are subchondral geodes and femoral head destruction that is disproportionately advanced relative to the acetabular involvement [10].

#### 1.2.2. Trauma

**Figure 9:**
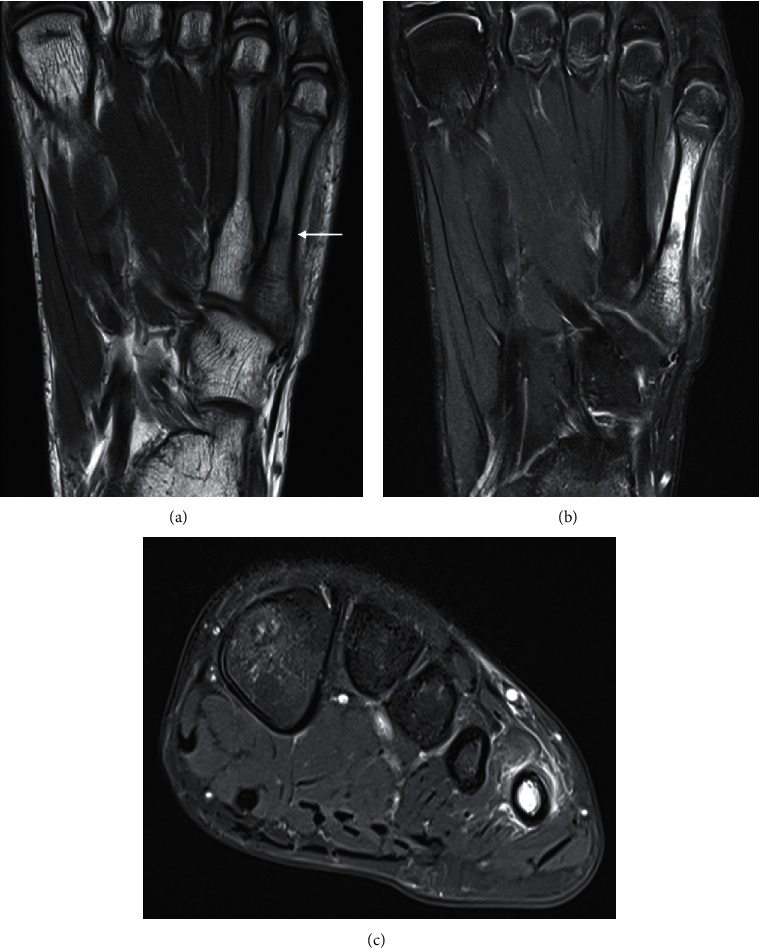
Stress response. Coronal PD (a), coronal (b), and axial PDFS (c) of a 13-year-old soccer player with persistent forefoot pain. There is diffuse marrow oedema signal centred in the 5^th^ metatarsal shaft with surrounding periosteal and soft tissue oedema. The linear signal at the medial border of the metatarsal shaft represents an incomplete fracture (a, arrow).

Fractures or stress responses can sometimes be mistaken for infection on imaging, particularly when a history of trauma is not provided. Both trauma and infection can have surrounding the soft tissue oedema signal. The presence of a hypointense fracture line on MRI helps to distinguish a fracture from osteomyelitis (Figure 9). In absence of a clear fracture line, the stress response occurs more commonly at the mid-diaphysis, whilst infection typically occurs in areas of the increased vascular supply, such as at the epiphysis or metaphysis [1]. The location of stress fractures is usually characteristic, such as the tibia, distal fibula, and metatarsal shafts.

#### 1.2.3. Neoplasm

**Figure 10:**
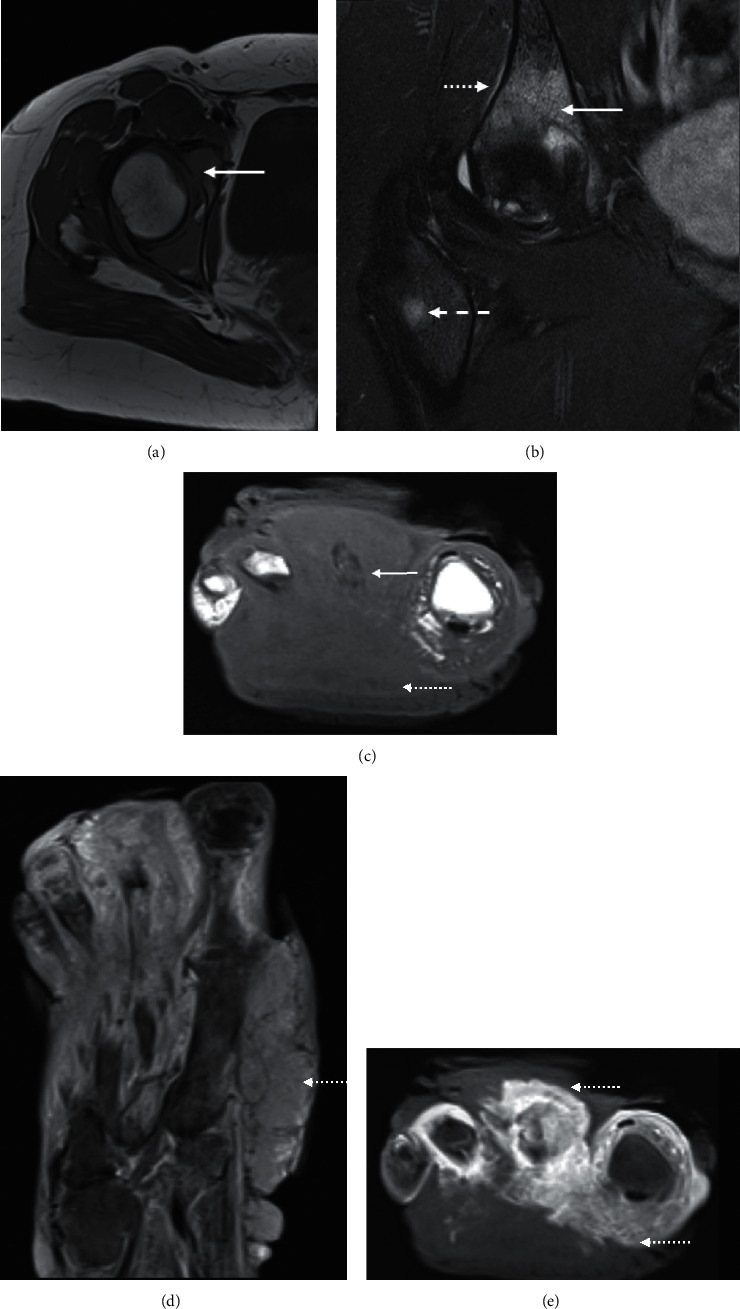
Metastases. Axial T1 (a) pelvis at the level of the hip joint of a 47-year-old female with metastatic breast cancer involving the right acetabulum. Ill-defined acetabular cortex is consistent with cortical breach of tumour and intraarticular extension (arrow). Tumour exhibits mass-effect that is usually readily apparent on MRI. Tumour is usually hyperintense in fluid-sensitive sequences which can mimic infection. Coronal PDFS (b) at the anterior acetabular margin with fluid-hyperintensity of the tumour breaching the acetabular cortex (arrow). Periosteal extension lateral pelvis (dotted arrow). A separate metastatic deposit also presents in the proximal right femoral shaft which helps clinch the diagnosis (dash). Axial T1 (c) and coronal STIR (d) and axial fat-saturated postcontrast T1 (e) of the right foot of a 38-year-old man with AIDS-related Kaposi sarcoma. The fungating, partially necrotic/enhancing mass (e) is centred in the forefoot, particularly involving the second metatarsal (arrow). The signal intensity of the involved bone mimics that of osteomyelitis, although infection should not be infiltrative and mass-forming (dotted arrows).

Neoplastic processes, either primary bone tumours or skeletal metastases, can also mimic infection. Tumours are usually mass-forming, and whilst this can be difficult to appreciate on CT or radiographs, this is usually evident on a T1 sequence [11] (Figure 10). It is important to assess for extraosseous extension of tumour and not confuse it with soft tissue inflammation (Figure 10(c)–10(e)).

**Figure 11:**
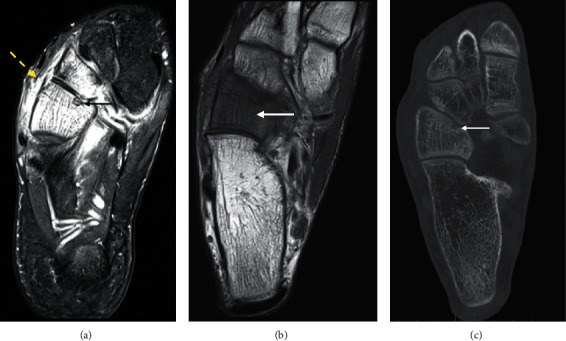
Cuboid osteoid osteoma in a 17-year-old presenting with a 2-month history of foot pain. Axial PDFS (a) of the foot shows diffuse marrow oedema-signal in the cuboid and lateral cuneiform with adjacent synovitis and soft tissue oedema. There is T1 marrow replacement of the cuboid ((b), arrow). A fluid-hyperintense nidus, best seen on the fluid-sensitive sequence, is noted in the medial border of the cuboid, with extension into the articular surface (arrow), accounting for the effusion and reactive synovitis (A yellow dash). The nidus is confirmed on the CT ((c), arrow), demonstrating central calcification, and distinguishes the diagnosis of osteoid osteoma from infection.

One specific neoplasm that can be confused with infection is osteoid osteoma. This is a benign osteoblastic neoplasm which is richly innervated and induces a florid host response by means of periostitis and marrow oedema signal [12]. In addition, up to 10% can be intra-articular which can incite marked synovitis and joint effusion (Figure 11). The clue to the correct diagnosis is the detection of a nidus at the epicentre of the marrow oedema signal, which is usually T1 hypointense and T2 hyperintense (Figure 11). The nidus can often be better appreciated on CT and can have a calcified or lucent matrix.

#### 1.2.4. Vasculopathy

**Figure 12:**
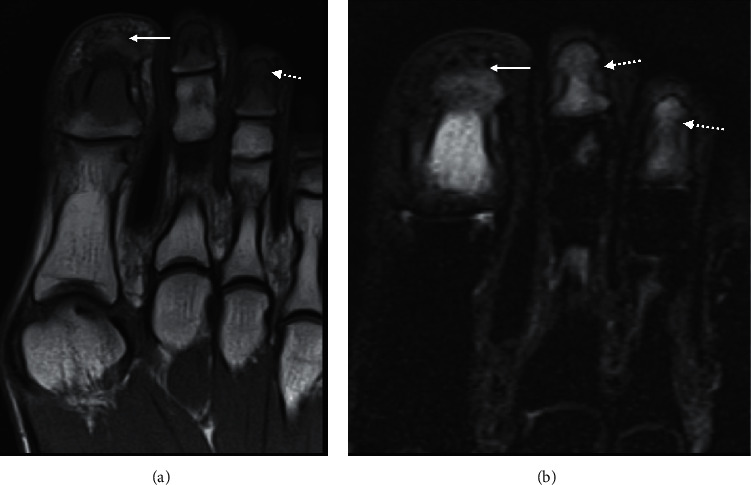
Coronal forefoot T1 (a) and STIR (b) sequence of a 45-year-old female with Raynaud phenomenon. There is an altered marrow signal (A dotted arrow) with oedema signal (b dotted arrow) in the distal phalanges involving noncontiguous bone. There is an impression of early tuft resorption involving the great toe (arrow), with an ill-defined cortex.

Raynaud phenomenon can affect the vasculature of the extremities by vasospasm and vasoconstriction following exposure to various stressors such as cold temperature. This can be associated with an abnormal marrow oedema-like signal limited to the distal phalanges of the hands or feet [13]. Similar changes have also been observed in patients with critical limb ischemia. Although the mechanism remains unclear, the signal changes may be due to tissue damage from ischaemic injury, oedema following reperfusion, or fibrosis from marrow necrosis. In these cases, noncontiguity of the marrow oedema-like signal involving multiple bones and absence of surrounding soft tissue abnormality is the key to accurate diagnosis (Figure 12).

#### 1.2.5. MRI Artefact: Failure of Fat Suppression

**Figure 13:**
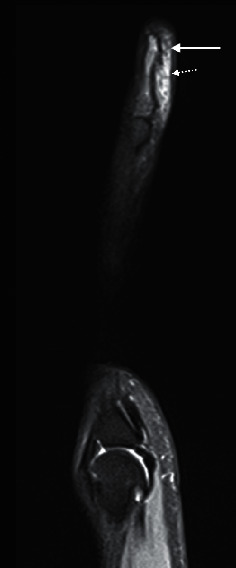
Artefact. Sagittal PDFS of the ring finger of a healthy volunteer demonstrating a failure of fat suppression at the tip of the finger resulting in a false impression of marrow hyperintensity in the distal phalanx. The adjacent subcutaneous tissue is also inadequately suppressed (dash).

The failure of fat suppression on MRI, manifesting as increased fluid-signal towards the extremities of the scan field, or in presence of metalware which can alter the uniformity of the magnetic field, is a potential artefactual pitfall. This can be distinguished from pathology, as the adjacent subcutaneous fat will also be involved or incompletely suppressed (Figure 13). Various techniques can be employed to minimise these artefacts, although this is beyond the scope of the manuscript [5].

#### 1.2.6. Inflammation

Inflammatory spondyloarthropathy comprises a group of chronic inflammatory rheumatologic conditions with a predilection for the axial skeleton [14, 15]. The sacroiliac joints are usually involved during the early course of the disease process. Early detection of sacroiliitis using a combination of radiologic and clinical findings is important since the condition usually responds well to disease-modifying therapies, which can prevent or slow the progression to structural damage.

**Figure 14:**
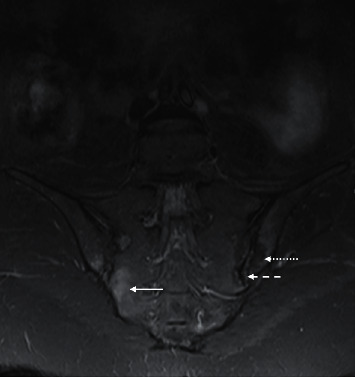
Sacroiliitis. Coronal STIR sequence of sacroiliac joints (SIJ) of a 34-year-old male with right acute on chronic and left chronic sacroiliitis. The region of the acute inflammatory process is denoted by the marrow oedema signal in the cartilaginous component of the right SIJ (arrow). Marrow fat infiltration (dotted arrow) adjacent to the left SIJ inferiorly implies prior inflammation. Irregular joint surface (dash) is consistent with erosion.

MRI manifestations include marrow oedema on both sides of the sacroiliac joints, usually involving the anteroinferior or cartilaginous articulation. In the early stages of the disease, joint effusion and marrow oedema signal can be more pronounced at the iliac border as the cartilage is thinner here. As the disease progresses, fatty infiltration in adjacent bone, erosion, and ankylosis can be observed (Figure 14). This inflammation, unlike infection, is confined to this space, does not cross anatomical borders, and is not associated with surrounding soft tissue oedema [14].

**Figure 15:**
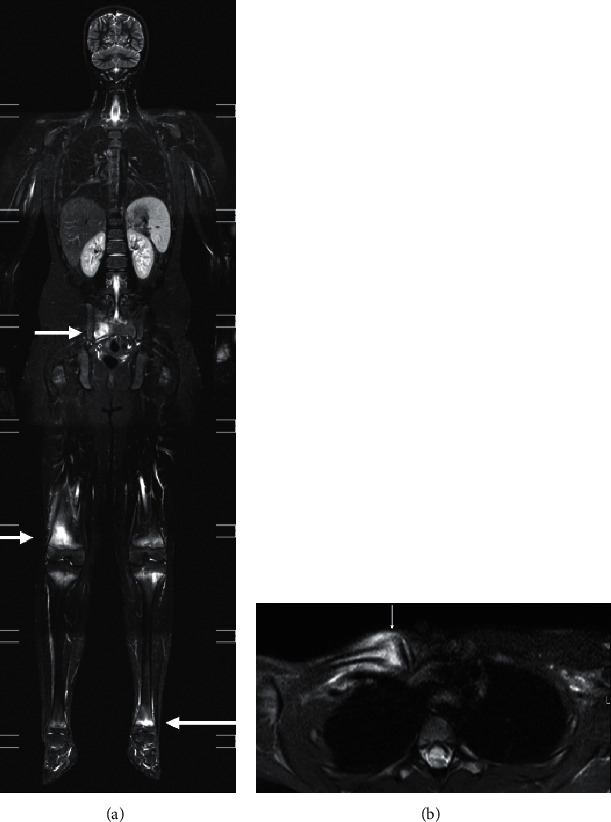
CRMO. Coronal whole-body MRI of an 8-year-old boy (a) with CRMO initially presenting with right knee pain. Note the multifocal involvement which included both knees, left ankle, and the sacrum (arrow). Axial STIR (b) of a different CRMO patient aged 7, with classic involvement of right medial clavicle with associated hyperostosis (arrow).

Chronic recurrent multifocal osteomyelitis (CRMO) is an idiopathic inflammatory disorder and shares similar appearances with subacute/chronic osteomyelitis [16]. Medial clavicular and multifocal involvement in children or adolescents is classically described. In suspected cases of CRMO, a whole-body MRI should be considered to look for other areas of bone involvement (Figure 15). CRMO is a diagnosis of exclusion after excluding other secondary causes for chronic OM.

**Figure 16:**
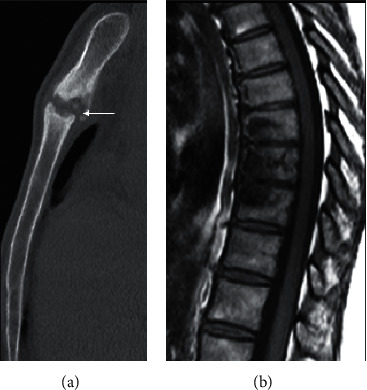
A 27-year-old female with SAPHO. There is hyperostosis and osteitis of the sternomanubrial junction on the sagittal CT (a). Contiguous involvement of multiple vertebral bodies anteriorly with sparing of the intervening disc space as demonstrated on sagittal T1 thoracic spine is typical of this condition (b).

SAPHO (synovitis, acne, pustulosis, hyperostosis, osteitis) is a rare condition affecting mostly young to middle-aged adults, representing an older demographic than the CRMO subset [17]. It may sometimes mimic osteomyelitis due to its bony involvement and osteitis. This is also usually polyostotic and classically affects the sternoclavicular joints, first rib, sternomanubrial joint, and the anterior aspect of the vertebral bodies (Figure 16).

#### 1.2.7. Iatrogenic Processes

**Figure 17:**
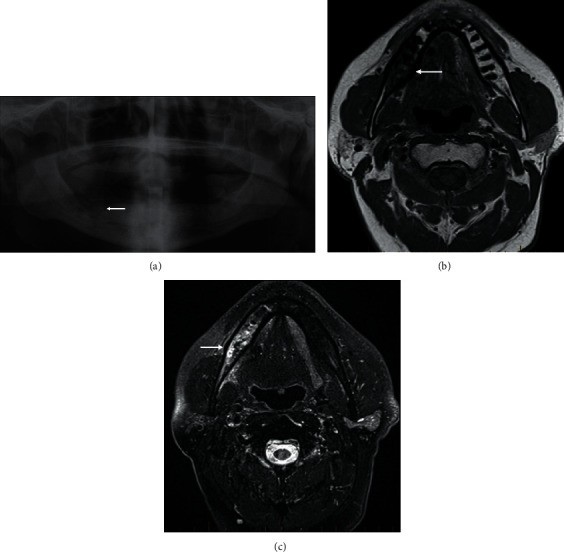
Osteoradionecrosis. Orthopantomogram (OPG, (a)) of a 74-year-old male with a history of T3 oropharyngeal tumour having undergone prior resection and chemoradiotherapy, complicated by osteoradionecrosis. There is ill-defined lytic lucency eroding the right mandibular body with background coarsened trabeculation and patchy sclerosis. Axial T1 (b) and T2FS (c) of a different patient with a previous history of radiotherapy for skin SCC, presenting with a nonhealing ulcer due to osteoradionecrosis. There are T1 hypointensity and fluid-hyperintensity in the body of the right mandible ((a) arrow). Mild surrounding soft tissue oedema ((b) arrow).

Radiation osteonecrosis can occur following radiotherapy (30–50 Gy) due to a combination of damage to osteoblasts, resulting in unopposed osteoclastic activity, and vascular injury [18]. There is an initial osteopenia or lysis, followed by a delayed and disorganised bony repair at least 3 years following radiotherapy, resulting in trabecular disorganisation and patchy marrow density (Figure 17(a)). On MRI, the initial acute changes can be associated with marrow and soft tissue oedema, and these can be difficult to distinguish from osteomyelitis or tumour recurrence (Figures 17(b) and 17(c)). Radiotherapy treatment timing, duration, dose, and field are useful parameters in this setting along with previous, baseline imaging for comparison, as radiation osteonecrosis will be confined within the field of radiotherapy even when the borders of the abnormal bone are poorly defined [19]. Associated osteolysis, osseous expansion, or fistula formation is usually more indicative of infection than osteonecrosis [19].

Postoperative findings and sterile collections can often be difficult to distinguish from infection and image interpretation can be further complicated by the presence of orthopaedic hardware which can lead to susceptibility and field inhomogeneity artefacts (Figure 13). The presence of metalware reduces the ability to adequately suppress the fat-signal, simulating marrow and soft tissue oedema. Whilst various MRI techniques have been employed for artefact reduction, nuclear medicine studies and, in particular, the white cell scan remains the most valuable imaging modality to assess for infection around a prosthesis [20].

**Figure 18:**
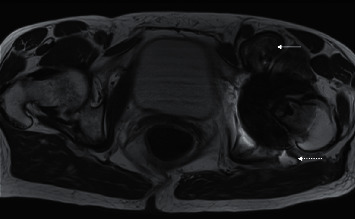
Adverse reaction to metallic debris (ARMD). A 70-year-old female with left hip resurfacing complicated by pseudotumour formation. Axial T2 pelvis with metal artefact reduction technique demonstrating distension of the left hip pseudocapsule posteriorly (dotted line) and a larger anterior pseudocapsule defect decompressing into the iliopsoas bursa (arrow). This thickened, T2-hypointense rim with synovial proliferation favours pseudotumour/ARMD over infection.

MRI plays a key role in the assessment of delayed-hypersensitivity reactions to arthroplasty-related metal products, known as pseudotumour or adverse reaction to metal debris. This can lead to aggressive soft tissue destruction if untreated. Imaging features include effusion with variable thickness of the synovial lining of synovial proliferation (Figure 18). It can be associated with perisynovial oedema and local nodal enlargement, mimicking infection. Increased synovial thickness and T1 and T2-hypointensity favour pseudotumour over infection [21].

#### 1.2.8. Neuropathy

**Figure 19:**
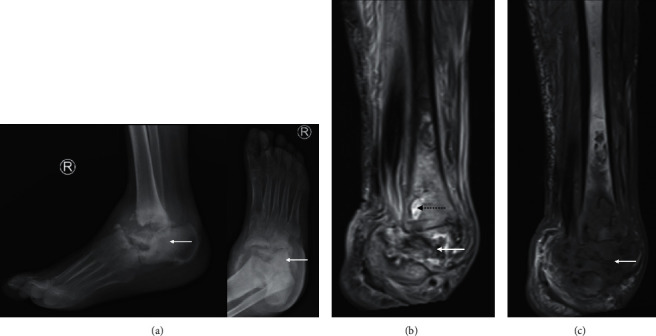
Lateral and oblique right foot radiographs (a) of a 47-year-old woman with type II diabetes and Charcot arthropathy involving the midfoot. Disorganised hindfoot with joint destruction and disorganisation (arrow). Note the absence of osteopenia that is typically observed in infection. Coronal STIR (b) and T1 (c) of a different patient with Charcot arthropathy with hindfoot destruction, hindfoot valgus, and dislocation. The contrast was not administered due to chronic renal failure. The talus is flattened and sclerotic (arrow). Note the fluid collection in the distal tibial shaft (dotted arrow) and the medial tibiotalar articulation. This patient had both neuropathic arthropathy complicated by chronic osteomyelitis with intraosseous abscess formation. At times, different conditions can preexist, and ultimately, tissue sampling may be required.

Neuropathic arthropathy most commonly occurs in patients with altered pain sensation and proprioception resulting in the accumulation of microtrauma [22, 23]. It commonly occurs in the feet of diabetic patients. In the acute stage, the foot may be painful and erythematous, followed by bone disorganisation, dislocation, secondary degeneration, and debris formation with preservation of bone density, also known as the “Five Ds” (Figure 19). Features that favour neuropathy over osteomyelitis are periarticular marrow oedema-like signal, joint subluxation, and geode formation, since neuropathy is predominantly an articular process. Sclerosis on radiographs and stability of findings over time favour Charcot neuropathy over infection. On the contrary, the contiguity of the marrow change with any ulcer or collection supports infection (Figures 1, 19(b), and 19(c). The areas affected by osteomyelitis may be more conspicuous on contrast-enhanced sequences. Various new techniques such as the use of diffusion-weighted imaging (DWI), dynamic postcontrast enhancement, and perfusion may provide further useful information although the utility of these more advanced techniques remains to be validated [22].

#### 1.2.9. Depositional Disease

**Figure 20:**
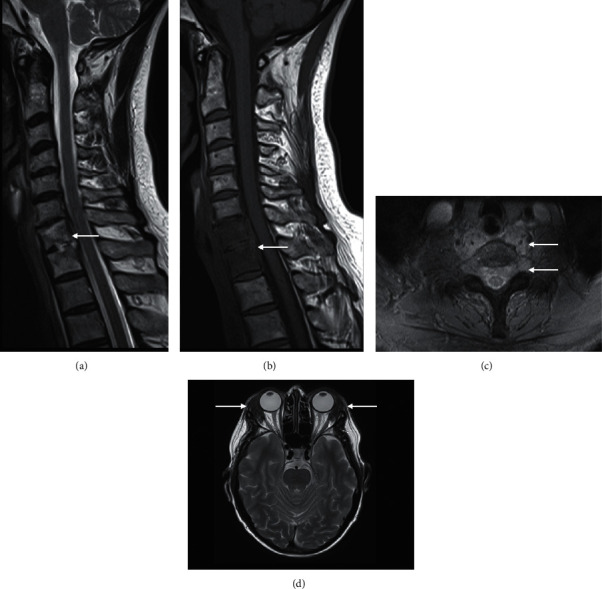
Primary amyloidosis. A 64-year-old woman with known primary amyloidosis of lacrimal glands, initially presenting with neck pain. Sagittal T2 (a), T1 (b), and axial T2FS (c) spine shows an infiltrative process involving the C7 and T1 vertebrae with an extraosseous extension of soft tissue into the epidural and prevertebral spaces (arrow). End plate irregularity is also present. Axial T2FS (c) at the level of C7 demonstrating epidural and prevertebral soft tissue infiltration (arrow). This case was difficult to distinguish from infection, ultimately requiring a biopsy for the final diagnosis of C7/T1 amyloid deposition. Axial T2 head at the level of the orbits (d) showed bilateral lacrimal gland enlargement secondary to amyloid deposition (arrow).

**Figure 21:**
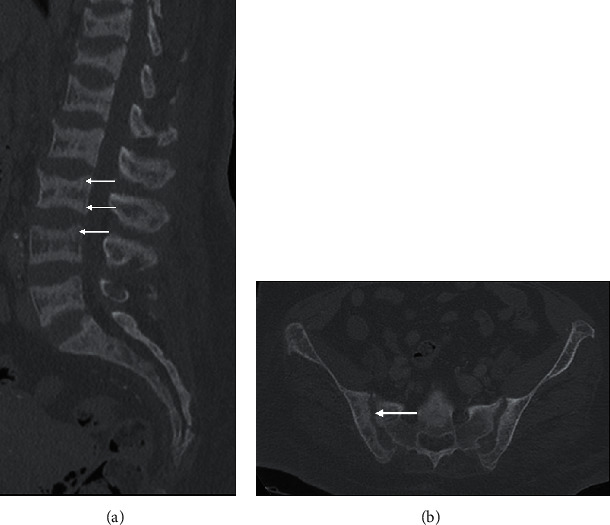
A 49-year-old female with end stage renal failure and tertiary hyperparathyroidism. Sagittal lumbar spine (a) with bone reconstructions shows rugger-jersey spine with sclerosis adjacent to end plates (arrow) whilst axial pelvis (b) demonstrates subperiosteal resorption of the SIJs (arrow).

Depositional disease such as amyloidosis can radiologically resemble an infection, ultimately requiring biopsy for diagnosis (Figure 20). In patients with chronic renal insufficiency, aggressive spondylosis can also occur due to amyloid deposition. Features of hyperparathyroidism such as sclerosis adjacent to end plates (rugger-jersey spine) and subperiosteal or subligamentous bone resorption can help clinch the diagnosis [24] (Figure 21).

**Figure 22:**
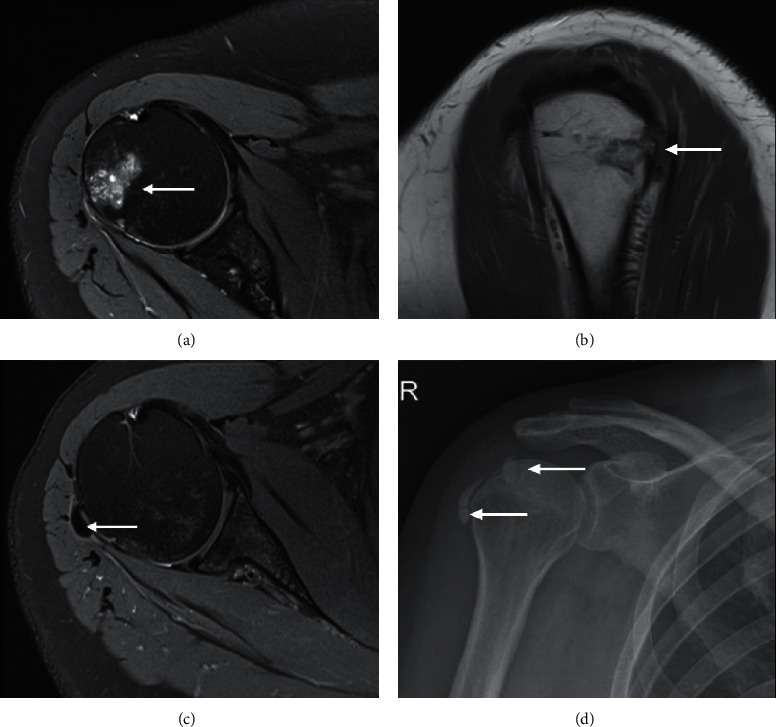
Calcific tendinitis in a 40-year-old female presenting with persistent shoulder pain. Axial PDFS (a) demonstrating marrow oedema signal in the humeral head at the greater tuberosity at teres minor tendon footprint. Sagittal PDFS (b) demonstrating corresponding cortical erosion. The MRI performed 5 months previously at the comparable plane (c) showed globular hypointense structure within the teres minor tendon footprint (arrow), confirming intraosseous migration of the calcific tendinitis in the most recent images. AP radiograph of the right shoulder (d) performed at the time of initial presentation also demonstrated calcium deposition in rotator cuff tendons. The calcium deposit is not well appreciated in the more recent study given the acute resorptive phase.

**Figure 23:**
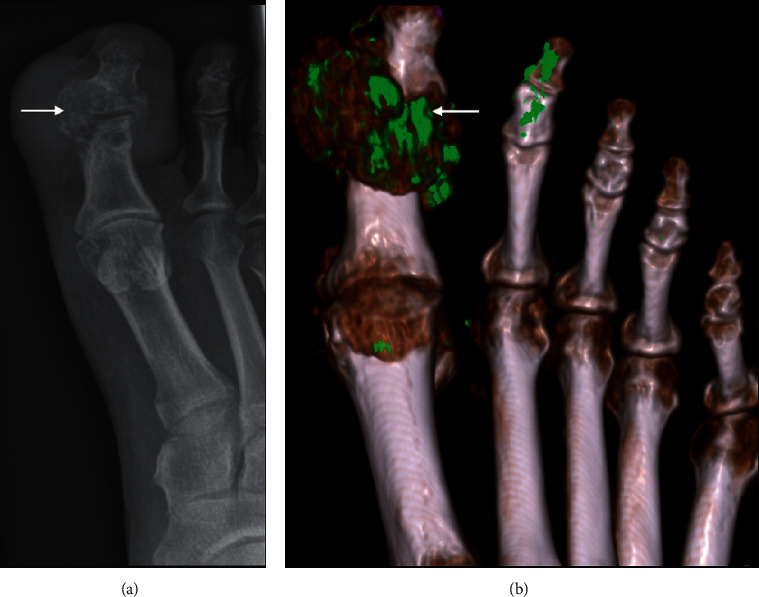
Gouty tophus in a 39-year-old man presenting with a swollen and erythematous toe which was clinically suspected to be an infection. X-ray showed 1^st^ MTPJ erosion at the medial border associated with dense soft tissue swelling (arrow). Dual-energy CT with material decomposition helped differentiate urate crystals (mapped as green, arrowed) from calcium (blue, not present in this case).

Microcrystals can deposit in periarticular or intraarticular soft tissue. This includes monosodium urate in gouty arthropathy and calcium hydroxyapatite deposition. Most cases are usually asymptomatic although the crystals can, at times, incite an inflammatory response and result in bone erosion, mimicking infection. In the acute phase, soft tissue swelling and joint effusion can be present. More chronic crystal deposits are usually T1 and T2 hypointense, without significant blooming artefact on susceptibility sequences that may otherwise suggest pigmented villonodular synovitis (PVNS). Calcific phosphate can deposit within tendons and can provoke an acute inflammatory response in the resorptive phase, during which the calcium can migrate into the adjacent bursa or bone, mimicking osteomyelitis (Figure 22). Calcium at this phase may be oedematous and difficult to depict on MRI. A radiograph can be useful for the assessment of soft tissue calcification which may not be apparent on MRI [25]. Dual-energy CT can also be used in distinguishing gout from other calcium deposition (Figure 23).

## 2. Conclusion

The imaging appearance of the bone and joint infections may be mimicked by many conditions. It is important to consider other differentials when a specific clinical scenario is discordant from the imaging findings. Whilst it is always important to consider the clinical picture and laboratory findings when interpreting radiologic findings, pertinent information may not always be available. An appreciation of the overlap of these differential diagnoses with infection and key differentiating factors equips the radiologist with the ability to make a significant contribution to diagnosis and appropriate, timely management.

## Data Availability

No new data were used to support this study.
